# Supplementary material for: Complex Regulation of Gamma-Hemolysin Expression Impacts Staphylococcus aureus Virulence
Source: Microbiol Spectr. 2023 Jun 22;11(4):e01073-23. doi: 10.1128/spectrum.01073-23 (PMC10434192; doi:10.1128/spectrum.01073-23)
Supplement: Supplemental file 1 — Supplemental material. Download spectrum.01073-23-s0001.docx, DOCX file, 3.7 MB [file spectrum.01073-23-s0001.docx]

**Supplementary methods and Results**

Complex regulation of gamma-hemolysin expression impacts *S. aureus* virulence

Mariane Pivard^a,*^, Isabelle Caldelari^b^, Virginie Brun^c,d^, Delphine Croisier^e^, Michel Jaquinod^c^, Nelson Anzala^e^, Benoît Gilquin^c,d^, Chloé Teixeira^a^, Yvonne Benito^f, g^, Florence Couzon^a^, Pascale Romby^b^, Karen Moreau^a^, François Vandenesch^a,f,g#^

^a^ – CIRI, Centre International de Recherche en Infectiologie, Université de Lyon, Inserm U1111, Université Claude Bernard Lyon 1, CNRS UMR5308, ENS de Lyon, Lyon, France.

^b^ - Architecture et Réactivité de l’ARN, Université de Strasbourg, CNRS, IBMC, Strasbourg, France

^c^ - Université Grenoble Alpes, Inserm, CEA, UMR BioSanté U1292, CNRS, CEA, FR2048, Grenoble, France

^d^ - Université Grenoble Alpes, CEA, LETI, Clinatec, Grenoble, France

^e^ - Vivexia, Dijon, France

^f^- Institut des agents infectieux, Hospices Civils de Lyon, Lyon, France.

^g^ - Centre National de Référence des Staphylocoques, Hospices Civils de Lyon, Lyon, France

^#^ - Address correspondence to: [francois.vandenesch@univ-lyon1.fr](mailto:francois.vandenesch@univ-lyon1.fr)

^*^ - Present address: Department of Infectious Diseases and Hospital Epidemiology, University Hospital

**INDEX:**

Text S1. Supplemental methods.

Figure S1. PVL, HlgABC and Hla contribute to *S. aureus* virulence during pneumonia in PVL-positive strain ST80.

Figure S2. *hlg*B does not have its own promoter, unlike *hlg*C.

Figure S3. Primer extension assays to probe the transcriptional start sites of *hlg*C and *hlg*B.

Figure S4. *hlg*B mRNA is a maturation product of the *hlg*CB transcript.

Figure S5. Pairwise sequence alignment: only few SNPs are detected in the *hlg*C upstream and coding regions of USA300, ST80, and PEN strains.

Figure S6. *hlg*B mRNA translation initiation does not require *hlg*C translation.

Figure S7. Secondary structure prediction of *hlg*C 5’UTR including 40 nucleotides from the coding sequence.

Table S1. Relative quantities of HlgC and HlgB in PEN wild-type and mutant strains by semi-quantitative proteomic analysis.

Table S2. Strains and plasmids used in this study.

Table S3. USA300, ST80, PEN and PEN HlgC<Q63X strains have similar growth after 8 h in CCY medium

Table S4. Oligonucleotides used in this study.

References

**SUPPLEMENTAL METHODS**

**Construction of mutant strains**

*Stop codon mutant*

Annealed *hlgC* spacer oligos (Table S4) were first inserted into the pnCasSA-BEC plasmid (pnCasSA-BEC-*hlg*C) by Golden Gate assembly (NEB). Then, pnCasSA-BEC-*hlg*C was electroporated into the *S. aureus* RN4220. The colonies were selected on TSB agar plates containing chloramphenicol (10 mg/L) at 30 °C. pnCasSA-BEC-*hlg*C plasmid isolated from the RN4220 strain was transformed into *S. aureus* PEN strain by electroporation. The colonies were also selected on TSB agar plates with chloramphenicol (10 mg/L) at 30 °C. The successful *hlgC* inactivation strain was confirmed by PCR and sequencing (Genome access on ENA with the accession number PRJEB61327), and the pnCasSA-BEC-*hlg*C was then cured by culturing the cells at 42 °C for 12 h without adding chloramphenicol.

*pMAD deletion*

Briefly, two DNA PCR fragments generated using oligonucleotides hlg525-*Sma*I with hlg2631-*Xho*I and hlg5916-*Xho*I with hlg7578-*Sal*I (Table S4), corresponding to the chromosomal DNA regions upstream *hlgA* and downstream *hlgB,* respectively, were cloned into the pMAD, an X-gal and thermo-sensitive plasmid (1). The resulting plasmid, pLUG1041, was electroporated into RN4220, and then into USA300 and ST80. For PEN, which is macrolide resistant, the plasmid pLUG1041 was modified by insertion of the chloramphenicol resistance gene *cat* from PC194 plasmid (2) into the multiple cloning sites. Transformants were grown at non-permissive temperature (37°C), to select cells in which the plasmid had been integrated into the chromosome by homologous recombination. To favor the second recombination event, a single colony was grown at 30°C for 10 generations and plated at 37°C overnight. Cells, which have lost the plasmid vector through a double crossing over event, were detected on X*gal* agar plates. PCR amplifications and sequencing were used to confirm the deletion of the *hlgACB* genes in the modified strains.

**Absolute quantification of HlgC and HlgB in clinical strains using targeted proteomics**

Targeted proteomics analyses were performed on a 6500 QTrap mass spectrometer (AB Sciex) operating in the selected reaction mode (SRM). Liquid chromatography (LC) separation was performed on an Ultimate 3000 system (Thermo Fischer) coupled to a C18 Kinetex™ column (2.6 µm, 100 Å, 2.1 mm, 10 cm) using a two-solvent system with solvent A (2% acetonitrile, 0.1% formic acid) and solvent B (80% acetonitrile, 0.1% formic acid). Peptide separation was performed over 40 min by applying a gradient from 2% to 32% solvent B in 30 min, and from 32% to 90% solvent B in 10 min, at a flow rate of 50 µL/min. Mass spectrometry data were acquired in positive mode with an ion spray voltage of 4200 V; curtain gas was used at 30 p.s.i. and the interface heater temperature was set to 320 °C. Collision exit, declustering, and entrance potentials were set to 37, 55, and 14 V, respectively. Collision energy was set to 31.4 for the YVSLINYLPK peptide and 29.2 for the SNFNPEFLSVLSHR peptide. Scheduled SRM acquisitions were performed with Q1 and Q3 quadrupoles operating at unit resolution, the acquisition time windows and target scan time were set to 120 sec and 1.2 sec, respectively. The SRM transitions monitored were:

605.4++/[y8] – 947.6+; 605.4++/[y7] – 860.5+; 605.4++/[y6] – 747.4+; 605.4++/[y5] – 634.4+ for the YVSLINYLPK peptide

609.4++/[y8] – 955.6+; 609.4++/[y7] – 868.5+; 609.4++/[y6] – 755.5+; 609.4++/[y5] – 642.4+ for the labeled YVSLINYLP[13C6, 15N2]K peptide

549.6+++/[y8] – 958.6+; 549.6+++/[y7] – 811.5+; 549.6+++/[y6] – 698.4+; 549.6+++/[y5] – 611.4+ for the SNFNPEFLSVLSHR peptide

553.0+++/[y8] – 968/6+; 553.0+++/[y7] – 821.5+; 553.0+++/[y6] – 708.4+; 553.0+++/[y5] – 621.4+ for the SNFNPEFLSVLSH[13C6, 15N4]R peptide

**Analyzing the 5’ end of *hlg*CB with the 5’-phosphate-dependent exonuclease Terminator™**

As previously described (3), 20 µg of total RNAs from USA300, ST80, and PEN strains extracted from 8 h cultures were incubated for 1 h at 30°C with or without 2 U of Terminator™ exonuclease (Lucigen). RNAs were purified by phenol-chloroform alcohol isoamyl extraction and absolute ethanol precipitation, and analyzed by Northern blot as described in the main manuscript.

**Relative-quantification by mass spectrometry**

HlgC and HlgB relative quantifications were obtained as previously described (4). Briefly, strains were cultured in 4 mL of CCY over night at 37°C with agitation, and then sub-cultured in 4 mL of CCY and incubated for 8 hours at 37°C with agitation. 1 mL of culture were collected, frozen, and stored at -80°C until denaturation step at 90°C for 1 hour. Then, samples were stored at -80°C until mass spectrometry preparation and analysis.

**Figure S1. PVL, HlgABC and Hla contribute to *S. aureus* virulence during pneumonia in PVL-positive strain ST80.** Kaplan-Meier survival curves for rabbits infected by endotracheal instillation of 9.49 to 9.6 log10 colony-forming units (CFU) /mL of ST80 wild type, ST80Δ*hla*, ST80Δ*luk*SF-PV, ST80 HlgC<Q63X or ST80 Δ*hla*Δ*luk*SF-PVΔ*hlg*ABC to induce necrotizing pneumonia. Log-rank (Mantel-Cox) test was used to compare mortality of mutants to that of wild type.


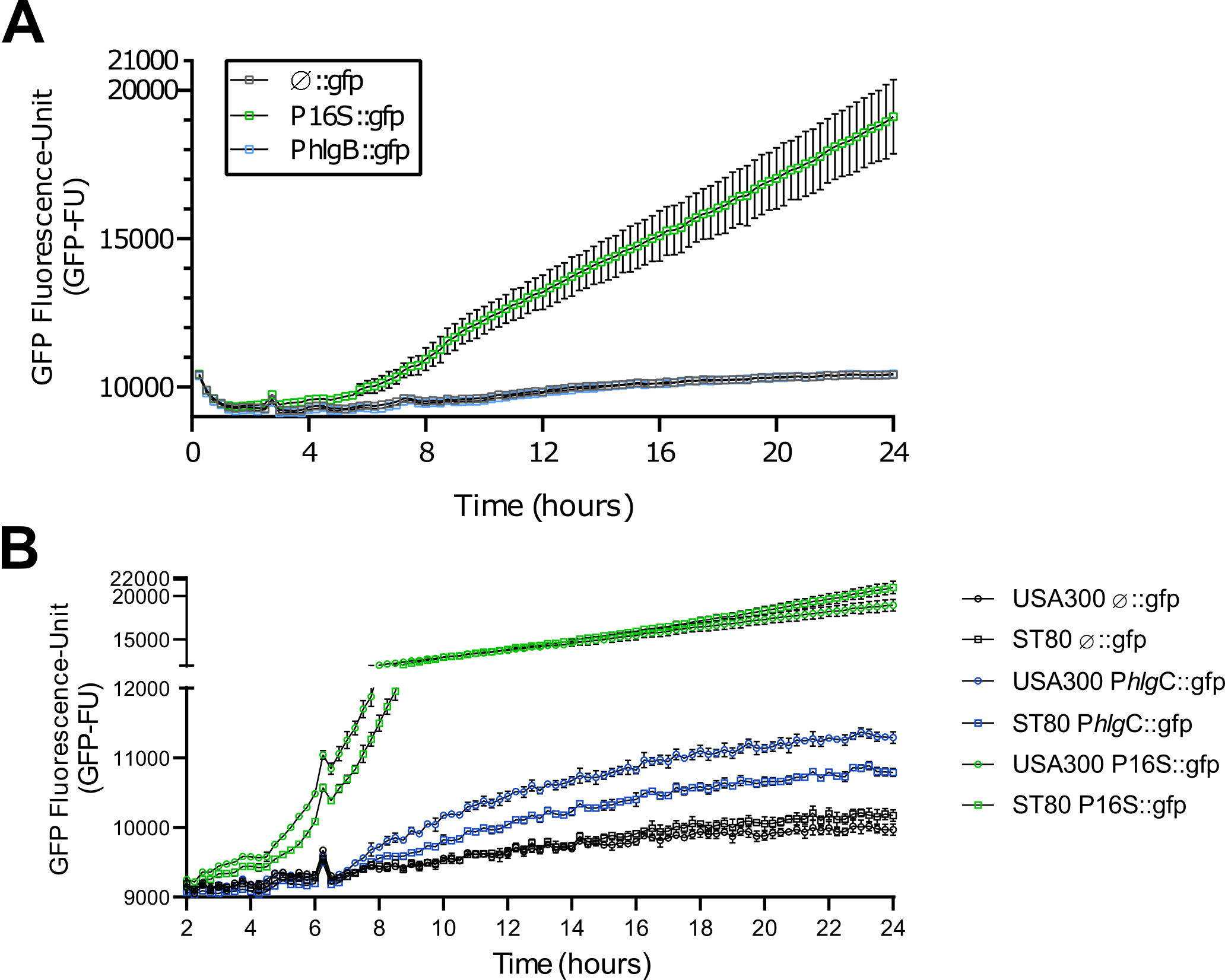


**Figure S2. *hlg*B does not have its own promoter, unlike *hlg*C**

(A) The existence of an *hlg*B-specific promoter within the *hlg*C gene was tested using transcriptional fusions in the ST80 strain. The 926bp region upstream *hlg*B and including *hlg*B ATG was cloned into the pACL-1484 plasmid in front of the *gfp* gene (P*hlg*B*::gfp*). The 16S rRNA promoter was used as positive control (P16S*::gfp*) and the plasmid with no insert as a negative control (⌀*::gfp*). (B) Same experiment with *hlg*C promoter (P*hlg*C) were conducted in both USA300 (circle) and ST80 (square) strains as another positive control of the system. Promoter activity was measured through GFP signal quantification (GFP Fluorescence Unit – GFP-FU) overtime, during a 24h kinetic, with a 15min interval time, in CCY with chloamphenicol. Mean with standard-error from biological triplicates are represented in grey for pACL-⌀, in green for pACL-P16S, and in blue for pACL-P*hlg*B in panel (A) and for pACL-P*hlg*C in panel (B).


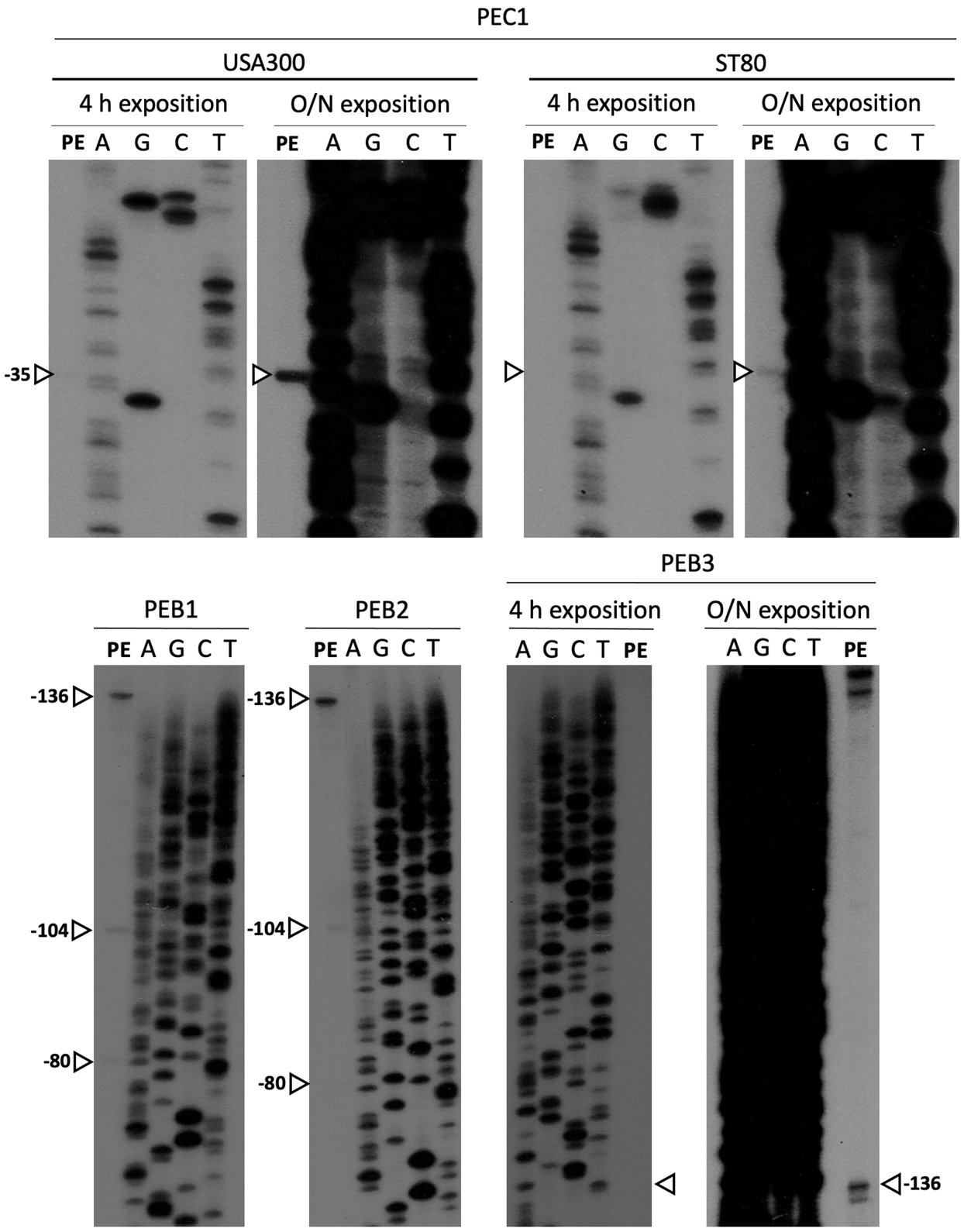


**Figure S3. Primer extension assays to probe the transcriptional start sites of *hlg*C and *hlg*B.**

Primer extension assays performed on total RNA of USA300 and ST80 strains using 5’-radiolabeled oligonucleotides complementary to *hlg*C (PEC1) and *hlg*B (PEB1, PEB2, and PEB3) sequences. Total RNAs were extracted from ST80 and USA300 strains after 8 h of growth in CCY medium. Radiolabelled primers and exposition times (4 h and overnight (O/N)) are mentioned above the gels. A, G, C, and T correspond to sequencing ladders, PE to primer extension, and the white arrows denote transcriptional start sites. The numbering of the transcriptional start sites is relative to *hlg*C and *hlg*B AUG initiation codon positions. Data are representative of at least two independent experiments.


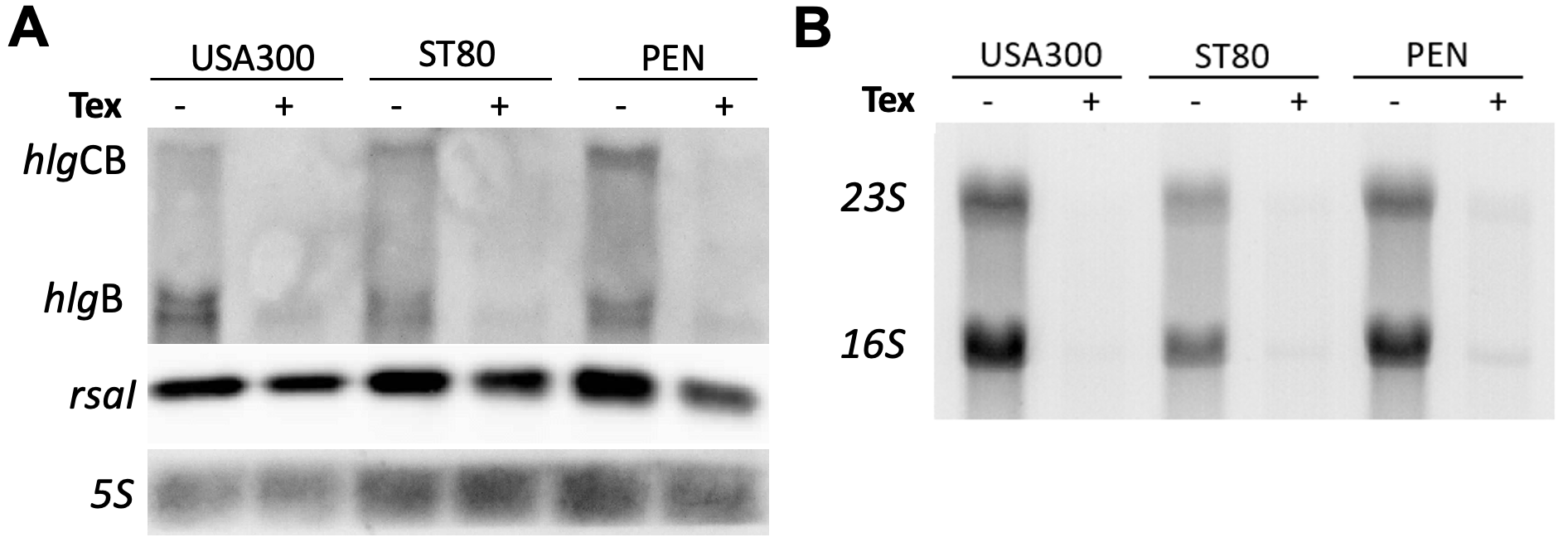


**Figure S4. *hlg*B mRNA is a maturation product of the *hlg*CB transcript**

Determination of the 5′ end status of *hlg*CB and *hlg*B. (A) Twenty micrograms of total RNA extracted after 8 h of growth in CCY were treated with the Terminator™ 5′-phosphate-dependent exonuclease (+ Tex). *hlg*CB and *hlg*B were probed with the *hlg*B probe, *rsa*I and 5S RNA were used as negative controls. (B) 16S and 23S, used as positive controls of Terminator™ 5′-phosphate-dependent exonuclease, were run on agarose gel and stained by ethidium bromide (EtBr).


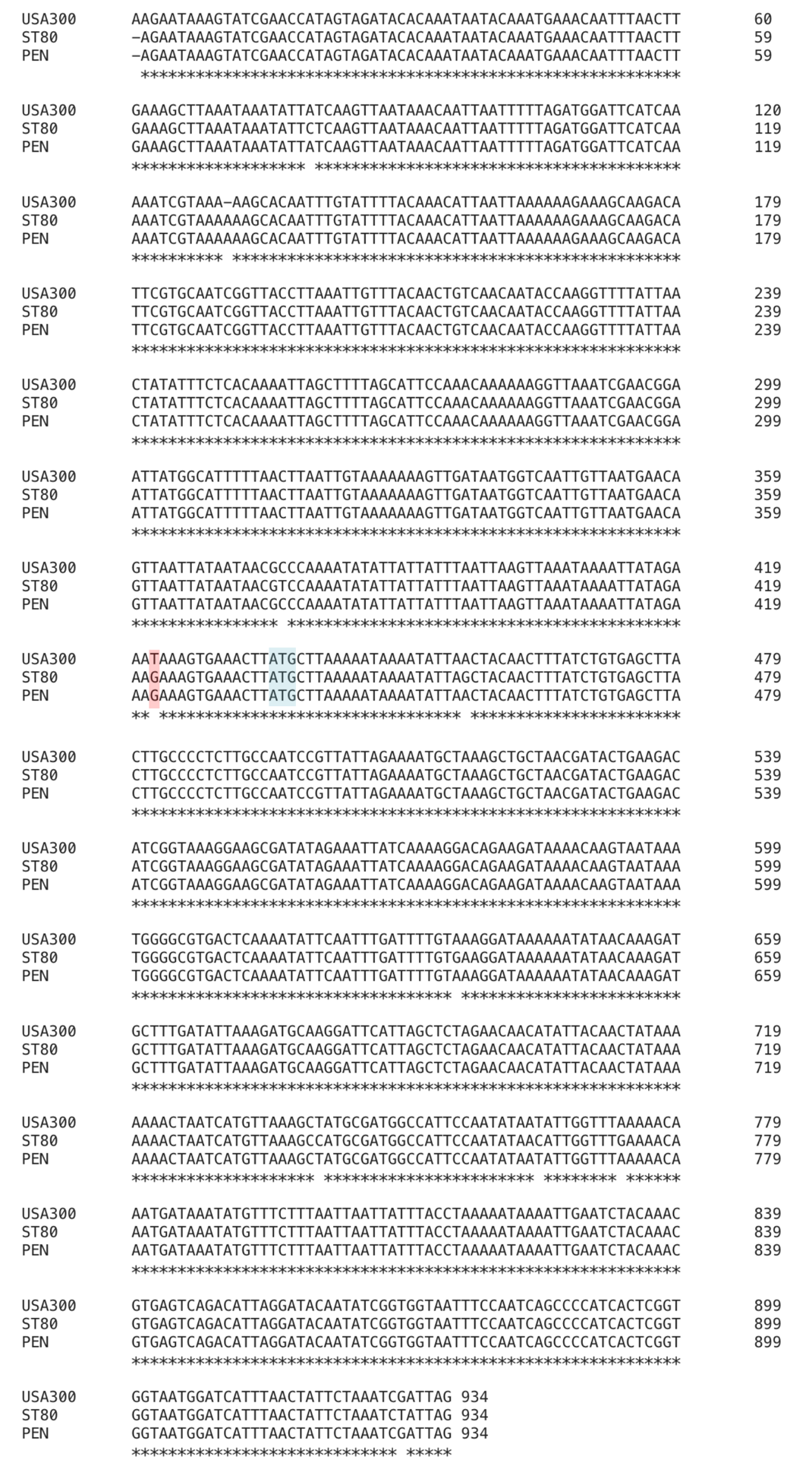


**Figure S5. Pairwise sequence alignment: only few SNPs are detected in the *hlg*C upstream and coding regions of USA300, ST80, and PEN strains.**

Alignment of 434 nts upstream and 500 nts downstream the start initiation codon (colored in light blue) of *hlg*C in USA300, ST80, and PEN strains. Stars represent matches and blanks indicate deletion/substitution or insertion. The SNP at position -13 from the start codon is highlighted in light red.

**
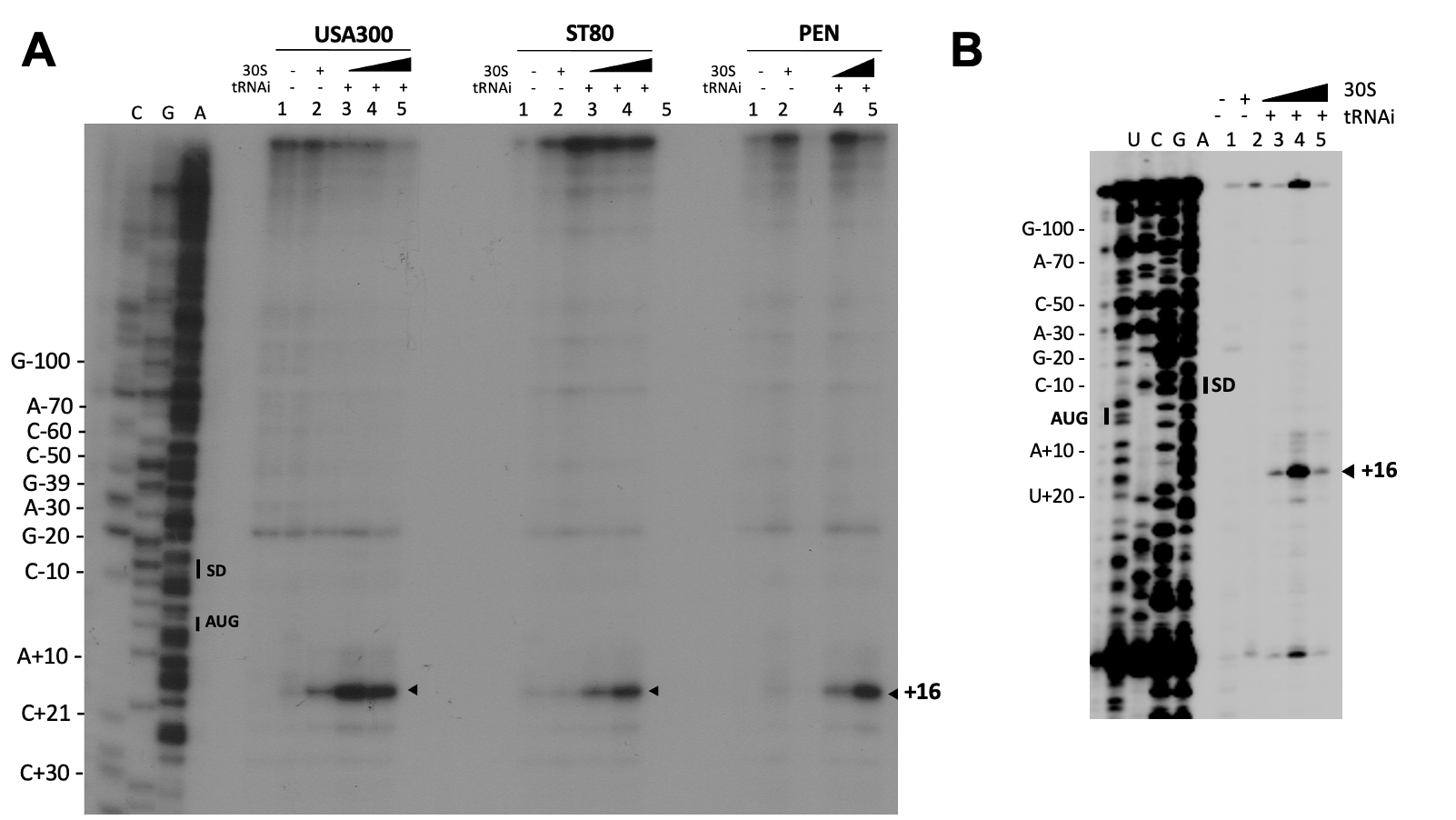
**

**Figure S6. *hlg*B mRNA translation initiation does not require *hlg*C translation**

Toe-print assays showing binding of *S. aureus* ribosomes on *hlg*CB mRNA (2007 nucleotides long) from USA300, ST80, and PEN strains (**A**) and *hlg*B (**B**) mRNAs (1159 nucleotides long) in *hlg*B upstream region. Lane 1: incubation control of mRNA alone; Lane 2: incubation control of mRNA with 30S subunits; Lane 3-5: formation of the ribosomal ternary complex containing mRNA (1 nM), the initiator tRNA_f_^Met^ (tRNAi, 1 µM), and increasing concentrations of 30S: 0.5 µM (Lane 3), 1 µM (Lane 4), and 2 µM (Lane 5). Lanes U, C, G, and A: sequencing ladders. The Shine and Dalgarno (SD) sequence, the initiation codon (AUG), and the toe-printing signal (+16) are indicated.


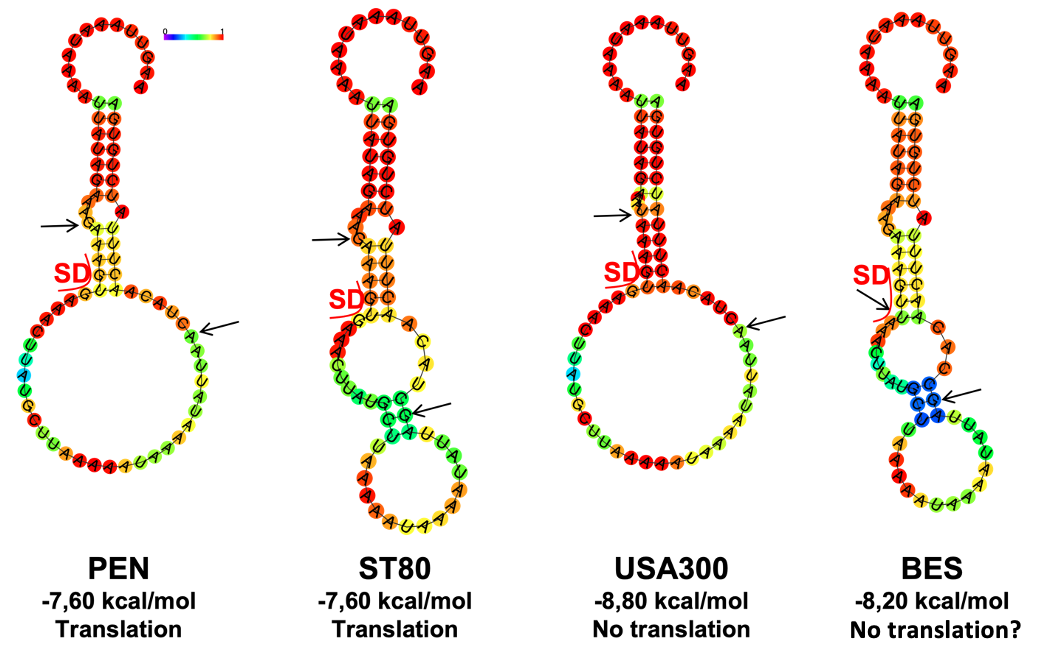


**Figure S7:** **Secondary structure prediction of *hlg*C 5’UTR including 40 nucleotides from the coding sequence.**

The prediction was done using the RNAfold web server from the Vienna RNA Websuite (5). The nucleotides are colored by base-pairing probabilities and for the unpaired region, the color denotes the probability of being unpaired. SD is for Shine and Dalgarno sequence, and arrows denote the nucleotide changes in the four strains. The minimum free energy corresponded to the main helix. *hlgC* mRNA is translated in PEN and ST80 while translation is impaired in USA300. “No translation?” is for unknown data.

**Supplementary Table I: Relative quantities of HlgC and HlgB in PEN wild-type and mutant strains by semi-quantitative proteomic analysis**

| Strains | HlgC | HlgB |
| --- | --- | --- |
| PEN WT | 1.5713 | 0.4922 |
| PEN <Q63X | 0 | 0.2222 |

**Supplementary Table II: Strains and plasmids used in the study**

|  | **Descriptions** | **References** |
| --- | --- | --- |
| Bacterial strains | | |
| *E. coli* strains | | |
| DH5α | Mutant *end*A1, *hsd*R17, *rec*A | Lab collection |
| *S. aureus* strains | | |
| RN4220 | Cloning intermediate | (6) |
| USA300 SF8300 | Community-acquired MRSA strain, *pvl*+, United States | (7) |
| USA300 pACL-Ø::*gfp* | USA300 SF8300 transformed with pACL1484 empty | This study |
| USA300 pACL-P*hlg*B::*gfp* | USA300 SF8300 transformed with pACL-P*hlg*B | This study |
| USA300 pACL-P*hlg*C::*gfp* | USA300 SF8300 transformed with pACL-P*hlg*C (U) | This study |
| USA300 pACL-P16S::*gfp* | USA300 SF8300 transformed with pACL-P16S | This study |
| USA300 SF8300 ∆*hlg* | Deletion of the *hlg* locus (*hlg*A and *hlg*CB) | (8) |
| USA300 ∆*hlg* pCN38::P3 | USA300 SF8300 ∆*hlg* complemented with the pCN38 with P3 promoter | This study |
| USA300 ∆*hlg* pCN38::P3::*hlg*CB (U) | USA300 SF8300 ∆*hlg* complemented with the pCN38 with P3 promoter and *hlgC*B sequence from USA300 SF8300 | This study |
| USA300 ∆*hlg* pCN38::P3::*hlg*CB (S) | USA300 SF8300 ∆*hlg* complemented with the pCN38 with P3 promoter and *hlgC*B sequence from ST80 | This study |
| USA300 ∆*hlg* pCN38::P3::*hlg*CB (P) | USA300 SF8300 ∆*hlg* complemented with the pCN38 with P3 promoter and *hlgC*B sequence from PEN | This study |
| ST80 | Community-acquired MRSA strain, *pvl*+, European | (9) |
| ST80 pACL-Ø::*gfp* | ST80 transformed with pACL1484 empty | This study |
| ST80 pACL-P*hlg*B::*gfp* | ST80 transformed with pACL-P*hlg*B | This study |
| ST80 pACL-P*hlg*C::*gfp* | ST80 transformed with pACL-P*hlg*C (S) | This study |
| ST80 pACL-P16S::*gfp* | ST80 transformed with pACL-P16S | This study |
| ST80∆*hlg* | Deletion of the *hlg* locus (*hlg*A and *hlg*CB) | This study |
| ST80∆*hla* | Deletion of the *hla* gene | This study |
| ST80∆*pvl* | Deletion of the *pvl* operon (*luk*S-PV and *luk*F-PV*)*) | This study |
| ST80 HlgC<Q63X | Premature stop codon in HlgC at the position 63 | This study |
| ST80∆*pvl*∆*hla*∆*hlg*ACB | Deletion of the *pvl* operon (*luk*S-PV and *luk*F-PV*)*), the *hla* gene and the *hlg* locus (*hlg*A and *hlg*CB) | This study |
| ST80 ∆*hlg* pCN38::P3 | ST80 ∆*hlg* complemented with the pCN38 with P3 promoter | This study |
| ST80 ∆*hlg* pCN38::P3::*hlg*CB (U) | ST80 ∆*hlg* complemented with the pCN38 with P3 promoter and *hlgC*B sequence from USA300 SF8300 | This study |
| ST80 ∆*hlg* pCN38::P3::*hlg*CB (S) | ST80 ∆*hlg* complemented with the pCN38 with P3 promoter and *hlgC*B sequence from ST80 | This study |
| ST80 ∆*hlg* pCN38::P3::*hlg*CB (P) | ST80 ∆*hlg* complemented with the pCN38 with P3 promoter and *hlgC*B sequence from PEN | This study |
| PEN426 (PEN) | Clinical isolate from severe community-acquired pneumonia patient, *pvl*- | (10) |
| PEN HlgC<Q63X | Premature stop codon in HlgC at the position 63 | This study |
| PEN ∆*hlg* | Deletion of the *hlg* locus (*hlg*A and *hlg*CB) | This study |
| PEN ∆*hlg* pCN38::P3 | PEN ∆*hlg* complemented with the pCN38 with P3 promoter | This study |
| PEN ∆*hlg* pCN38::P3::*hlg*CB (U) | PEN ∆*hlg* complemented with the pCN38 with P3 promoter and *hlgC*B sequence from USA300 SF8300 | This study |
| PEN ∆*hlg* pCN38::P3::*hlg*CB (S) | PEN ∆*hlg* complemented with the pCN38 with P3 promoter and *hlgC*B sequence from ST80 | This study |
| PEN ∆*hlg* pCN38::P3::*hlg*CB (P) | PEN ∆*hlg* complemented with the pCN38 with P3 promoter and *hlgC*B sequence from PEN | This study |
| Clinical isolates from severe community-acquired pneumonia patients, *pvl*- | | (10) |
| BAC434 | CC5-MSSA |  |
| BAN867 | CC15-MSSA |  |
| BEL175 | CC398-MSSA |  |
| BOF419 | CC45-MSSA |  |
| BRG113 | CC12-MSSA |  |
| CHA146 | CC398-MSSA |  |
| CHO435 | CC5-MSSA |  |
| COP340 | CC1-MSSA |  |
| GRJ356 | CC45-MSSA |  |
| LAA284 | CC30-MSSA |  |
| LAC295 | CC398-MSSA |  |
| LOE344 | CC398-MSSA |  |
| MAF251 | CC30-MSSA |  |
| MAS222 | CC398-MSSA |  |
| PIA965 | CC8-MSSA |  |
| SID287 | CC398-MSSA |  |
| SUD370 | CC5-MSSA |  |
| VIS047 | CC1-MRSA |  |
| ZAF193 | CC45-MSSA |  |
| Clinical isolates from severe community-acquired pneumonia patients, *pvl*+ | |  |
| ALA547 | CC80-MRSA |  |
| ARR376 | CC5-MRSA |  |
| BES288 | CC15-MSSA |  |
| CAB419 | CC30-MSSA |  |
| DOM365 | CC121-MSSA |  |
| DUC355 | CC30-MSSA |  |
| ERG344 | CC80-MRSA |  |
| FAL003 | CC8-MRSA |  |
| GAJ281 | CC121-MSSA |  |
| GOH148 | CC152-MSSA |  |
| MEN612 | CC1-MSSA |  |
| MRN242 | CC152-MSSA |  |
| MSO304 | CC88-MRSA |  |
| NEJ118 | CC80-MRSA |  |
| NIA469 | CC152-MSSA |  |
| PIR088 | CC121-MSSA |  |
| QUM434 | CC121-MSSA |  |
| RIP333 | CC121-MSSA |  |
| ROJ208 | CC15-MSSA |  |
| TOM044 | CC30-MSSA |  |
| Plasmids | | |
| *in vitro* transcription | | |
| pUC-T7::*hlg*CB (U) | Plasmid used for T7 transcription of *hlg*CB from USA300 SF8300 | This study |
| pUC-T7::*hlg*CB (S) | Plasmid used for T7 transcription of *hlg*CB from ST80 |  |
| pUC-T7::*hlg*CB (P) | Plasmid used for T7 transcription of *hlg*CB from PEN |  |
| pUC-T7::*hlg*B | Plasmid used for T7 transcription of *hlg*B |  |
| Transcriptional fusion with GFP | | |
| pACL1484 | Promoterless *gfpuvr* shuttle vector | (11) |
| pACL-P*hlg*B | The 929nt upstream and including *hlg*B start codon cloned in front of the *gfppuv* gene | This study |
| pACL-P*hlg*C (U) | The 434nt upstream and including *hlg*C start codon from USA300 SF8300 cloned in front of the *gfppuv* gene |  |
| pACL-P*hlg*C (S) | The 434nt upstream and including *hlg*C start codon from ST80 cloned in front of the *gfppuv* gene |  |
| pACL-P*hlg*C (P) | The 434nt upstream and including *hlg*C start codon from PEN cloned in front of the *gfppuv* gene |  |
| pACL-P16S | 16S rRNA gene promoter cloned in front of the *gfppuv* gene | (12) |
| HlgC inactivation | | |
| pnCasSA-BEC | *S. aureus* base editing vector, Km^r^, Cm^r^ | (13) |
| pnCasSA-BEC-*hlg*C | pnCasSA-BEC plasmid with *hlg*C spacer | This study |
| Overexpression of hlgCB | | |
| pCN38 | Shuttle vector for *E. coli* and *S. aureus* | (14) |
| pCN38::P3 | pCN38 with P3 promoter | This study |
| pCN38::P3::*hlg*CB (U) | pCN38 with P3 promoter and *hlg*CB sequence from USA300 SF8300 |  |
| pCN38::P3::*hlg*CB (S) | pCN38 with P3 promoter and *hlg*CB sequence from ST80 |  |
| pCN38::P3::*hlg*CB (P) | pCN38 with P3 promoter and *hlg*CB sequence from PEN |  |
| In frame deletion | | |
| pMAD | Thermosensitive origin of replication, constitutively expressed *bgal* gene | (1) |
| pMAD::*hlg* | Plasmid to delete the *hlg* locus (*hlg*A and *hlg*CB) | (8) |
| pMAD::*hlg*-Cm | Plasmid to delete the *hlg* locus (*hlg*A and *hlg*CB) with Chloramphenicol resistance | This study |

**Supplementary Table III: USA300, ST80, PEN and PEN HlgC<Q63X strains have similar growth after 8 h in CCY medium**

| Strains | R1 | R2 | R3 | Means |
| --- | --- | --- | --- | --- |
| USA300 | 8.99 | 8.71 | 8.04 | 8.58 |
| ST80 | 8.92 | 8.14 | 8.81 | 8.62 |
| PEN | 9.10 | 9.04 | 8.82 | 8.98 |
| PEN <Q63X | 9.56 | 8.88 | 8.79 | 9.07 |

OD_600_ measurements for USA300, ST80, PEN and PEN HlgC<Q63X (PEN<Q63X) strains after 8 h culture in CCY. Three biological replicates (R1 to R3) have been performed and means of OD_600_ for each strain are mentioned in the column Means.

**Supplementary Table IV: Oligonucleotides used in the study**

| **Name** | **Sequence 5' - 3'** |
| --- | --- |
| ***hlg*C inactivation** | |
| hlgC-cas9-PEN-F | GAAATATTCAATTTGATTTTGTAA |
| hlgC-cas9-PEN-R | AAACTTACAAAATCAAATTGAATA |
| ***hlg* deletion** |  |
| hlg525-SmaI | TATCCCGGGACCCTATGC |
| hlg2631-XhoI | ATACTCGAGTTGTAAGCGTTTTCG |
| hlg5916-XhoI | AAACTCGAGTAACTTATCAACGTG |
| hlg7578-SalI | TATGTCGACCAAAAGTTACAAGC |
| **DIG probes** | |
| 5S-T7-R | **TAATACGACTCACTATAGGG**GATTTGTCATTTGCCTGGC |
| 5S-F | GTAAGTTATTTTGTCTGGTGGCTATAGC |
| hlgA-T7-R | **TAATACGACTCACTATAGGG**CGTTTGCTAGTAATGTCTTGTG |
| hlgA-F | CAGCAACTTTAGCAGTTGGT |
| hlgC-T7-R | **TAATACGACTCACTATAGGG**TGGAATGGCCATCGCATAG |
| hlgC-F | CTGAAGACATCGGTAAAGG |
| hlgB-T7-R | **TAATACGACTCACTATAGGG**CATAACTTTTATCTTTGATG |
| hlgB-F | GTCAAATCATCCGTTGCTA |
| RsaI-T7-R | **TAATACGACTCACTATAGGG**GCACGTGCTAGCCGACAAATA |
| RsaI-F | TAACAGGGGGAGCGATTAAA |
| **Primer extension** | |
| PEC1 | GGATTGGCAAGAGGGGCAAG |
| PEB1 | CAGAAAGTAATAATAATGCC |
| PEB2 | ATTTGACTAATTTATTCATT |
| PEB3 | GACCTCGTATTTCACAGTATAG |
| **Promoter activiy** | |
| PhlgC-EcoRI-F | GCGCGAATTCAGAATAAAGTATCGAACC |
| PhlgC-XbaI-R | GCGCTCTAGAGCATAAGTTTCACTTTATTTC |
| PhlgB-PuvII-F-USA | GCGCCAGCTGAACTACAACTTTATCTGTGAG |
| PhlgB-PuvII-F-ST80 | GCGCCAGCTGAGCTACAACTTTATCTGTGAG |
| PhlgB-KpnI-R | GCGCGGTACCATCAATTCTGTCCTTTCACC |
| pALC-F | AAATACCGCATCAGGCGCCA |
| pALC-R | CATCACCTTCACCCTCTCCA |
| ***in vitro* transcription** | |
| pUCT7-hlgC-StuI-F | TATAGGCCTAAGTTAAATAAAATTATAGAA |
| pUCT7-hlgB-StuI-F | TATAGGCCTAAAAGATCAACGCATTATGGC |
| pUCT7-hlgB-BamHI-R | TATGGATCCAAAACACGGTCGTCACAATTA |
| **Reverse transcription for toe-print** | |
| hlgC-R | ATCGCTTCCTTTACCGATGTC |
| hlgB-R | TAAAGTAACTTTGTCATCGAC |
| **Overexpression of *hlg*CB in pCN38** | |
| *hlgCB*-PstI-F | GGCCTGCAGAATTAAGTTAAATAAAATTATAG |
| *hlgCB*-BamHI-R | GGCGGATCCCTAAGATAATAAATCAAAAC |
|  |  |
| Restriction site sequences are underlined, the T7 promoter sequence is in bold. | |

**References:**

1. Arnaud M, Chastanet A, Débarbouillé M. 2004. New vector for efficient allelic replacement in naturally nontransformable, low-GC-content, gram-positive bacteria. Appl Environ Microbiol 70:6887–6891.

2. Horinouchi S, Weisblum B. 1982. Nucleotide sequence and functional map of pC194, a plasmid that specifies inducible chloramphenicol resistance. J Bacteriol 150:815–825.

3. Desgranges E, Barrientos L, Herrgott L, Marzi S, Toledo-Arana A, Moreau K, Vandenesch F, Romby P, Caldelari I. 2022. The 3′UTR-derived sRNA RsaG coordinates redox homeostasis and metabolism adaptation in response to glucose-6-phosphate uptake in Staphylococcus aureus. Molecular Microbiology 117:193–214.

4. Pivard M, Bastien S, Macavei I, Mouton N, Rasigade J-P, Couzon F, Youenou B, Tristan A, Carrière R, Moreau K, Lemoine J, Vandenesch F. 2023. Targeted proteomics links virulence factor expression with clinical severity in staphylococcal pneumonia. Frontiers in Cellular and Infection Microbiology 13.

5. Gruber AR, Lorenz R, Bernhart SH, Neuböck R, Hofacker IL. 2008. The Vienna RNA websuite. Nucleic Acids Res 36:W70-74.

6. Nair D, Memmi G, Hernandez D, Bard J, Beaume M, Gill S, Francois P, Cheung AL. 2011. Whole-genome sequencing of Staphylococcus aureus strain RN4220, a key laboratory strain used in virulence research, identifies mutations that affect not only virulence factors but also the fitness of the strain. J Bacteriol 193:2332–2335.

7. Diep BA, Palazzolo-Ballance AM, Tattevin P, Basuino L, Braughton KR, Whitney AR, Chen L, Kreiswirth BN, Otto M, DeLeo FR, Chambers HF. 2008. Contribution of Panton-Valentine leukocidin in community-associated methicillin-resistant Staphylococcus aureus pathogenesis. PLoS One 3:e3198.

8. Perret M, Badiou C, Lina G, Burbaud S, Benito Y, Bes M, Cottin V, Couzon F, Juruj C, Dauwalder O, Goutagny N, Diep BA, Vandenesch F, Henry T. 2012. Cross-talk between Staphylococcus aureus leukocidins-intoxicated macrophages and lung epithelial cells triggers chemokine secretion in an inflammasome-dependent manner. Cell Microbiol 14:1019–1036.

9. Vandenesch F, Naimi T, Enright MC, Lina G, Nimmo GR, Heffernan H, Liassine N, Bes M, Greenland T, Reverdy M-E, Etienne J. 2003. Community-acquired methicillin-resistant Staphylococcus aureus carrying Panton-Valentine leukocidin genes: worldwide emergence. Emerging Infect Dis 9:978–984.

10. Gillet Y, Tristan A, Rasigade J-P, Saadatian-Elahi M, Bouchiat C, Bes M, Dumitrescu O, Leloire M, Dupieux C, Laurent F, Lina G, Etienne J, Vanhems P, Argaud L, Vandenesch F, PVL pneumonia study group. 2021. Prognostic factors of severe community-acquired staphylococcal pneumonia in France. Eur Respir J 58:2004445.

11. Wolz C, Pöhlmann-Dietze P, Steinhuber A, Chien Y-T, Manna A, Van Wamel W, Cheung A. 2000. Agr-independent regulation of fibronectin-binding protein(s) by the regulatory locus sar in Staphylococcus aureus. Molecular Microbiology 36:230–243.

12. Nonfoux L, Chiaruzzi M, Badiou C, Baude J, Tristan A, Thioulouse J, Muller D, Prigent-Combaret C, Lina G. 2018. Impact of Currently Marketed Tampons and Menstrual Cups on Staphylococcus aureus Growth and Toxic Shock Syndrome Toxin 1 Production In Vitro. Appl Environ Microbiol 84:e00351-18.

13. Gu T, Zhao S, Pi Y, Chen W, Chen C, Liu Q, Li M, Han D, Ji Q. 2018. Highly efficient base editing in Staphylococcus aureus using an engineered CRISPR RNA-guided cytidine deaminase. Chem Sci 9:3248–3253.

14. Charpentier E, Anton AI, Barry P, Alfonso B, Fang Y, Novick RP. 2004. Novel cassette-based shuttle vector system for gram-positive bacteria. Appl Environ Microbiol 70:6076–6085.
